# Brief Consent Methods Enable Rapid Enrollment in Acute Stroke Trial: Results From the TICH-2 Randomized Controlled Trial

**DOI:** 10.1161/STROKEAHA.121.035191

**Published:** 2021-12-01

**Authors:** Zhe Kang Law, Jason P. Appleton, Polly Scutt, Ian Roberts, Rustam Al-Shahi Salman, Timothy J. England, David J. Werring, Thompson Robinson, Kailash Krishnan, Robert A. Dineen, Ann Charlotte Laska, Philippe A. Lyrer, Juan Jose Egea-Guerrero, Michal Karlinski, Hanne Christensen, Christine Roffe, Daniel Bereczki, Serefnur Ozturk, Jegan Thanabalan, Ronan Collins, Maia Beridze, Alfonso Ciccone, Lelia Duley, Angela Shone, Philip M. Bath, Nikola Sprigg

**Affiliations:** Stroke Trials Unit (Z.K.L., J.P.A., P.S., P.M.B., N.S.), University of Nottingham, United Kingdom.; Vascular Medicine, Division of Medical Sciences and GEM, Royal Derby Hospital Centre (T.J.E.), University of Nottingham, United Kingdom.; Radiological Sciences (R.A.D.), University of Nottingham, United Kingdom.; Nottingham Clinical Trials Unit (L.D.), University of Nottingham, United Kingdom.; Research and Innovation (A.S.), University of Nottingham, United Kingdom.; Department of Medicine (Z.K.L.), National University of Malaysia.; Department of Surgery, Division of Neurosurgery (J.T.), National University of Malaysia.; Stroke, University Hospitals Birmingham NHS Foundation Trust, United Kingdom (J.P.A.).; Clinical Trials Unit, London School of Hygiene and Tropical Medicine, United Kingdom (I.R.).; Centre for Clinical Brain Sciences, University of Edinburgh, United Kingdom (R.A.-S.S.).; Stroke Research Centre, UCL Queen Square Institute of Neurology, London, United Kingdom (D.J.W.).; Department of Cardiovascular Sciences and NIHR Biomedical Research Centre, University of Leicester, United Kingdom (T.R.).; Stroke, Nottingham University Hospitals NHS Trust, United Kingdom (K.K., P.M.B., N.S.).; NIHR Nottingham Biomedical Research Centre, United Kingdom (R.A.D.).; Department of Clinical Sciences, Karolinska Institute Danderyd Hospital, Sweden (A.C.L.).; Department of Neurology and Stroke Center, University Hospital Basel and University of Basel, Switzerland (P.A.L.).; NeuroCritical Care Unit, Virgen del Rocio University Hospital, Seville, Spain (J.J.E.-G.).; Institute of Psychiatry and Neurology, Warsaw, Poland (M.K.).; Department of Neurology, Bispebjerg Hospital and University of Copenhagen, Denmark (H.C.).; Stroke Research, School of Medicine, Keele University, Newcastle-Under-Lyme, United Kingdom (C.R.).; Department of Neurology, Semmelweis University, Budapest, Hungary (D.B.).; Selcuk University Faculty of Medicine, Department of Neurology, Konya, Turkey (S.O.).; Age Related Health Care/Stroke-Service, Tallaght University Hospital, Dublin, Republic of Ireland (R.C.).; The First University Clinic of Tbilisi State Medical University, GA (M.B.).; Neurology and Stroke Unit, Poma Hospital, ASST di Mantova, Mantua, Italy (A.C.).

**Keywords:** cerebral hemorrhage, humans, informed consent, logistic models, lost to follow-up, tranexamic acid

## Abstract

**Background:**

Seeking consent rapidly in acute stroke trials is crucial as interventions are time sensitive. We explored the association between consent pathways and time to enrollment in the TICH-2 (Tranexamic Acid in Intracerebral Haemorrhage-2) randomized controlled trial.

**Methods:**

Consent was provided by patients or by a relative or an independent doctor in incapacitated patients, using a 1-stage (full written consent) or 2-stage (initial brief consent followed by full written consent post-randomization) approach. The computed tomography-to-randomization time according to consent pathways was compared using the Kruskal-Wallis test. Multivariable logistic regression was performed to identify variables associated with onset-to-randomization time of ≤3 hours.

**Results:**

Of 2325 patients, 817 (35%) gave self-consent using 1-stage (557; 68%) or 2-stage consent (260; 32%). For 1507 (65%), consent was provided by a relative (1 stage, 996 [66%]; 2 stage, 323 [21%]) or a doctor (all 2-stage, 188 [12%]). One patient did not record prerandomization consent, with written consent obtained subsequently. The median (interquartile range) computed tomography-to-randomization time was 55 (38–93) minutes for doctor consent, 55 (37–95) minutes for 2-stage patient, 69 (43–110) minutes for 2-stage relative, 75 (48–124) minutes for 1-stage patient, and 90 (56–155) minutes for 1-stage relative consents (*P*<0.001). Two-stage consent was associated with onset-to-randomization time of ≤3 hours compared with 1-stage consent (adjusted odds ratio, 1.9 [95% CI, 1.5–2.4]). Doctor consent increased the odds (adjusted odds ratio, 2.3 [1.5–3.5]) while relative consent reduced the odds of randomization ≤3 hours (adjusted odds ratio, 0.10 [0.03–0.34]) compared with patient consent. Only 2 of 771 patients (0.3%) in the 2-stage pathways withdrew consent when full consent was sought later. Two-stage consent process did not result in higher withdrawal rates or loss to follow-up.

**Conclusions:**

The use of initial brief consent was associated with shorter times to enrollment, while maintaining good participant retention. Seeking written consent from relatives was associated with significant delays.

**Registration:**

URL: https://www.isrctn.com; Unique identifier: ISRCTN93732214.

Obtaining consent in a timely manner is a major challenge for investigators in hyperacute stroke trials.^1,2^ In these studies, the intervention has to be delivered in a short therapeutic time window to be effective. On the other hand, obtaining consent is difficult when patients lack capacity and no relatives or people with power of attorney are available to provide consent on their behalf.^1,2^ The COVID-19 pandemic has further complicated the consent process due to physical distancing precautions. Furthermore, patients or their representatives may also be overwhelmed by the acute scenario and unable to comprehend the information provided or the rationale for taking part in a clinical trial.^2^ Simplifying the consent process by providing concise but pertinent information to patients and their relatives may improve and shorten time to recruitment in acute stroke trials.

TICH-2 (Tranexamic Acid in Intracerebral Haemorrhage-2) endeavored to minimize time to enrollment by using a 2-stage pathway, where consent was sought initially from patients or their legal representatives using a brief information sheet followed by a full consent after randomization. We aimed to explore the characteristics of patients enrolled using 1- and 2-stage consent, the acceptability, and the effects on time to randomization. We hypothesized that the use of brief information sheets would reduce time to randomization.

## Methods

### Data Availability Statement

The trial data can be shared upon reasonable request to the corresponding author and the trial steering committee.

TICH-2 was an international prospective multicenter, double-blind, randomized placebo-controlled trial testing the efficacy and safety of intravenous tranexamic acid in patients with acute spontaneous intracerebral hemorrhage (ICH) within 8 hours of symptom onset. Details of the trial were published previously.^3–5^ The consent-related procedures were developed in partnership with stroke survivors, some of whom were members of the trial steering committee. The information sheets and consent forms were designed according to the principles outlined in the Medicines for Human Use (Clinical Trials Regulations) 2004 and European Clinical Trials Directive (EC2001/20) and based on templates provided by the UK Health Research Authority.^6^ The consent procedures were approved by each participating country or center’s ethics review committee.

This analysis is reported in accordance with the Strengthening the Reporting of Observational Studies in Epidemiology statement (Supplemental Methods S1 and S2).^7^

### Definitions

A personal legal representative is a person acting as a legal representative to an incapacitated patient by virtue of their relationship with the patient. A personal legal representative is usually a relative but could be a close friend who may be aware of the patients’ likely wishes if a relative was not available. A professional legal representative is a doctor or nominated health care professional unconnected to trial who acts as a patient’s legal representative to provide consent. A professional legal representative must not be involved in the trial management, be an investigator or part of the trial team, or be under the direction of the trial investigator.^8^ Proxy consent is a consent given by a personal or professional legal representative. For brevity purpose in this article, personal legal representative is referred to as a relative and professional legal representative as a doctor.

### Consent Pathways

The consent pathways consisted of 1-stage patient and relative consent, 2-stage patient and relative consent, and 2-stage doctor consent.

### One-Stage Patient Consent

In the 1-stage patient consent pathway, eligible patients were given the full-version patient information sheet. This was a 4-page information sheet (2474 words; Supplemental Methods S3) that explained the condition, the purpose of the trial, the drug being studied, randomization, blinding, follow-up assessments, possible benefit and harm, alternative treatments, withdrawal from the trial, data confidentiality and governance, dissemination of trial results, and complaint procedures. Patients were then required to provide full written informed consent by writing their initials adjacent to 7 relevant statements on the consent form and provide a full signature, name, and date. Patients with capacity were required to sign the form themselves. If handwriting was not possible or legible due to arm weakness or use of the nondominant hand, a third person acted as a witness by signing the form.

### One-Stage Relative Consent

For incapacitated patients, a relative was approached for proxy consent using the full-version legal representative information sheet (2668 words) and consent form with similar details as the full patient version detailed above (Supplemental Methods S4).

### Two-Stage Patient Consent

When it was deemed not feasible to seek full written informed consent due to a short therapeutic time window, patients provided initial consent by signing a brief information sheet, followed by full written informed consent at the earliest subsequent opportunity after enrollment. The brief information sheet consisted of only one page of information (288 words; Supplemental Methods S5) that explained the condition (ICH), treatment (tranexamic acid or placebo), blinding, and that there would be an additional computed tomography (CT) scan after 24 hours. The patients were required to sign only once and did not need to initial the statements as in full consent.

### Two-Stage Relative Consent

A 2-stage process using an initial brief legal representative information sheet (1 page, 290 words; Supplemental Methods S6) with similar details as the brief patient version could be used in seeking consent in incapacitated patients, followed later by full consent.

### Two-Stage Doctor Consent

When the patient lacked capacity and there was no relative available, an independent doctor was approached to provide proxy consent. The independent doctor was part of the clinical team caring for the patient and should not have received prior trial-related training. Information relating to the trial was provided to the doctor, and if he/she agreed for the patient’s inclusion in the trial, a brief information sheet was signed. Subsequently, a full written informed consent was sought from patients or their relatives as soon as practicable. Notably, doctors did not provide full consent.

The Figure describes the consent pathways in TICH-2. Different countries had slightly different consent pathways, versions of documents in different languages. All countries permitted the 1-stage patient consent, while there were variations in permission to use the other consent pathways (Table 1).

**Table 1.**
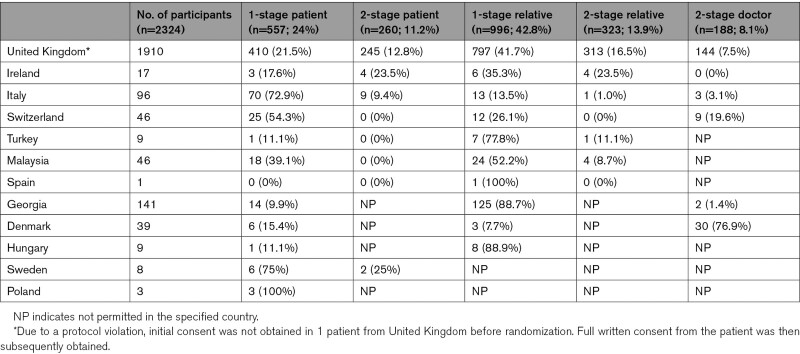
Types of Initial Consent in Each Country

**Figure.**
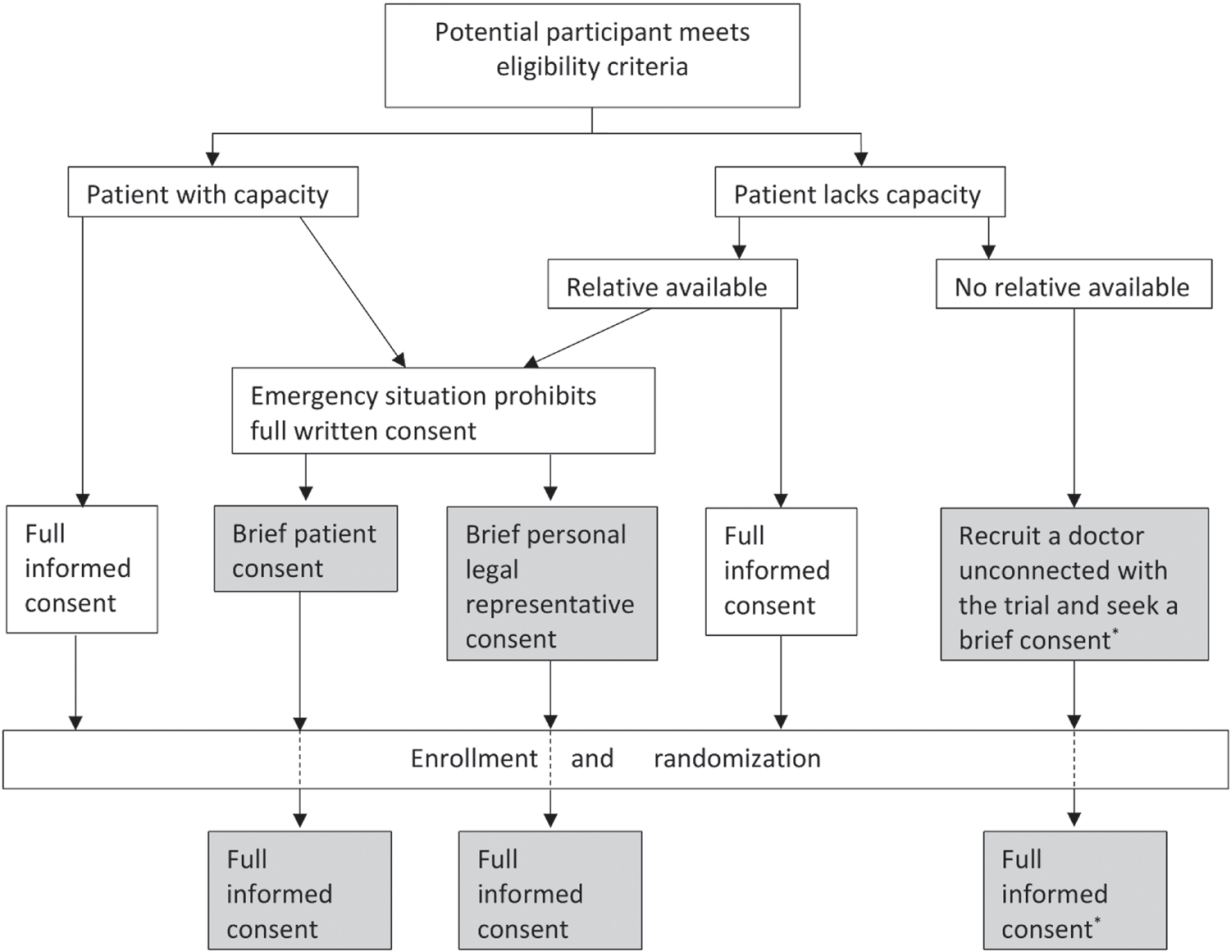
**Consent process in the TICH-2 trial (Tranexamic Acid in Intracerebral Haemorrhage-2).** *Patient who lacked capacity with no relatives present was discussed with an independent doctor who acted as a professional legal representative. Full consent was sought later from the patient (if regained capacity) or a relative, if available. Two-stage pathways are marked gray.

We retrospectively calculated the fog index, which assesses readability and estimates the level of education needed to understand the text on the first reading.^9^ The fog indices of the brief patient and legal representative information sheets were 7.8 and 8.0, respectively, and 11.4 and 12.3 for full patient and legal representative information sheet. A fog index of 8.0 indicates that a 13- or 14-year old would be able to understand at first reading, while text with a fog index of 12.0 can be understood by a 17- or 18-year old.

### Outcomes

We explored the time to enrollment, defined as CT-to-randomization time, according to different consent pathways. We specifically explored whether consent pathways were associated with onset-to-randomization time of ≤3 hours. This time window was chosen as a meta-analysis of over 40 000 patients with traumatic hemorrhage and postpartum hemorrhage suggested that tranexamic acid is only beneficial when given within 3 hours.^10^ Furthermore, most hematoma growth occurs within 3 hours.^11^ We compared follow-up completion and withdrawal rates according to types of initial consent as a surrogate marker for acceptability.

### Statistics

Baseline characteristics and outcomes were compared using χ^2^ tests for categorical variables and Mann-Whitney *U* and Student *t* tests for continuous variables as appropriate. Time to enrollment for different consent pathways was compared using χ^2^ tests for categorical variables and Kruskal-Wallis tests for medians. Multivariable logistic regression, with adjustment of variables with *P*<0.1 on univariate analysis, was performed to identify factors associated with onset-to-randomization times of ≤3 hours. In addition, we performed sensitivity analyses including only countries using 2-stage consent. A *P* of <0.05 was considered statistically significant, and 95% CIs are given. Analyses were performed using the Statistical Package for the Social Sciences, version 26 (IBM, Armonk, NY).

## Results

Two thousand three hundred twenty-five patients were recruited from 12 countries between March 2013 and September 2017. Only 817 patients (35.1%) gave self-consent by using 1-stage (557; 24%) or 2-stage consents (260; 11.2%). The majority of patients (1507; 64.8%) were enrolled by proxy consent provided by a relative (1-stage consent in 996 [42.8%] and 2-stage consent in 323 [13.9%]) or by a doctor (188; 8.1%). For 1 patient from the United Kingdom, written consent was not given at the time of randomization, and this was reported as a protocol violation; full consent was subsequently given by the patient.

Of the 771 patients who provided a brief consent initially (260 patient, 323 relative, and 188 doctor consents), follow-on full consent was given in all but 105 patients (13.6%) before hospital discharge. Reasons for not obtaining follow-on consent were death (n=38), patient lacked capacity and no relative available (n=38), discharged (n=8), repatriated (n=13), no reason given (n=6; Table S1). Only 2 patients, for whom a relative provided initial brief consent, declined to give further consent and withdrew from the trial before discharge. A further 2 patients who provided follow-on full consent withdrew before the day 90 follow-up. There was no significant difference in the number of patients lost to follow-up and in withdrawals at day 90 between the different consent pathways (Table S2).

Patients who were recruited following proxy consent were older, more likely to be female, and had higher National Institutes of Health Stroke Scale scores (median, 16 versus 7), lower Glasgow Coma Scale scores (median, 14 versus 15), severe aphasia (42.1% versus 2.9%), intraventricular hemorrhage (36.2% versus 24.2%), and larger hematoma volumes (mean, 30.2 versus 12.7 mL), but there was no significant difference in onset-to-CT time (Table 2). Patients who were recruited via proxy consent had more hematoma expansion (30.7% versus 21.9%), higher mortality (14.5% versus 2.7%) at day 7, and more death and disability (67.6% versus 30.5%) at day 90.

**Table 2.**
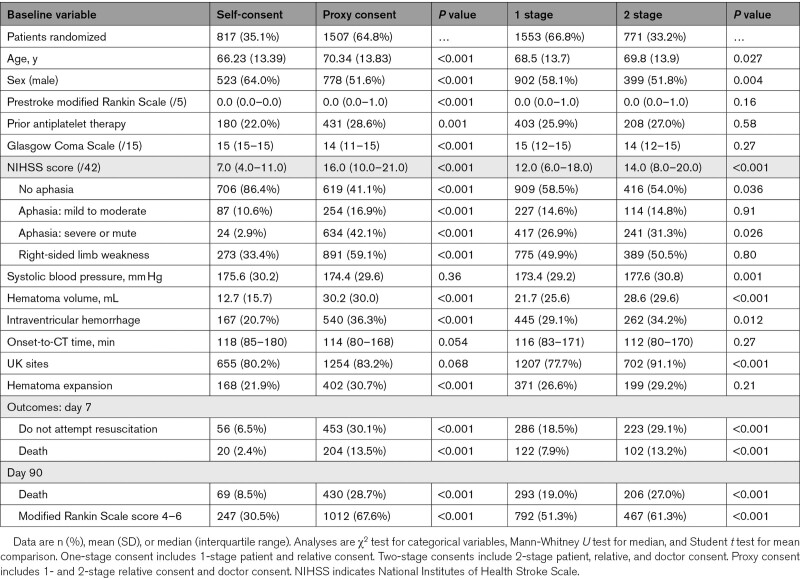
Characteristics and Outcome of Patients According to Consent Type

Similarly, patients who were recruited using 2-stage consent were older, more likely to be female, had higher National Institutes of Health Stroke Scale (median, 14 versus 12), more likely to have severe aphasia (31.3% versus 26.9%), intraventricular hemorrhage (34.2% versus 29.1%), larger hematoma volume (mean, 28.6 versus 21.7 mL), and were more likely to be recruited in the United Kingdom (Table 2). Patients recruited using 2-stage consent had higher mortality at day 7 (27% versus 19%) and more death and disability at day 90 (61.3% versus 51.3%).

Using 2-stage doctor and the 2-stage patient consent resulted in the shortest enrollment time (median CT-to-randomization time, 55 minutes for both) followed by the 2-stage relative (69 [43–110] minutes), the 1-stage patient (75 [48–124] minutes), and the 1-stage relative consent (90 [56–155]; Kruskal-Wallis test *P*<0.001; Table 3). The onset-to-treatment time was shortest with the 2-stage doctor consent (200 [149–259] minutes) compared with 267 (193–364) minutes for the 1-stage relative consent (the longest time to treatment). There was no significant difference in time from randomization to treatment between the consent pathways.

**Table 3.**
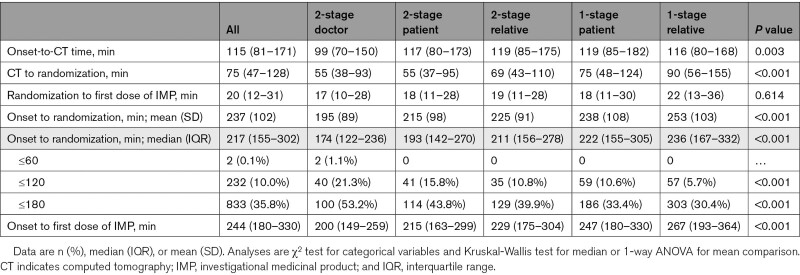
Enrollment Time of Patients According to Consent Pathways

Apart from consent pathways, other factors associated with an onset-to-randomization time of ≤3 hours include higher National Institutes of Health Stroke Scale, higher systolic blood pressure, shorter onset-to-CT time, and recruitment from the United Kingdom (Table S3). Multivariable logistic regression showed that factors independently associated with onset-to-randomization time of ≤3 hours include higher National Institutes of Health Stroke Scale, higher SBP, shorter onset-to-CT time, recruitment from the United Kingdom, and use of 2-stage patient and relative consent (adjusted odds ratio, 1.89 [95% CI, 1.49–2.39]; *P*<0.001; Table 4, model 1) and 2-stage doctor consent (adjusted odds ratio, 2.29 [1.52–3.47]; *P*<0.001; Table 4, model 2). Conversely, relative consent reduced the odds ratio of randomization ≤3 hours of onset (adjusted odds ratio, 0.10 [95% CI, 0.03–0.34]; *P*<0.001; Table 4, model 3). Sensitivity analysis excluding countries that did not recruit participants using 2-stage consent (Spain and Hungary, n=10) yielded similar results (Table S4).

**Table 4.**
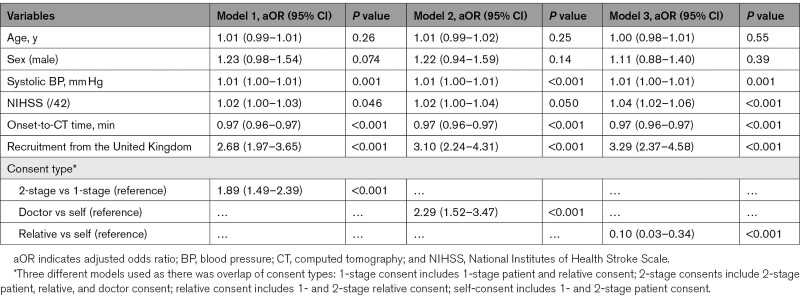
Multivariable Logistic Regression of Factors Associated With Time to Randomization of ≤3 h

## Discussion

In this post hoc analysis, we found that 2-stage consent using brief information sheets reduced the time to randomization by ≈20 minutes. CT-to-randomization time was the shortest with the 2-stage doctor or patient consent and longest for consent given by relatives. Apart from shortening onset-to-CT time with improvement of local stroke pathways, the use of the rapid consent pathway may be one of the most important approaches to improving time to enrollment for trials of treatments for acute ICH, as well as more generally in all acute stroke trials.

The brief information sheet, developed in consultation with our patient, carer, and public representatives, was concise but contained pertinent information, which allowed patients to decide to proceed with the trial while more information was given later. The shorter text and better readability reduced reading time and enabled easier understanding. In addition, time used to sign the consent form was shortened with the brief consent, as only one signature was required instead of 7 sets of initials and one signature in the full consent. Our approach in developing and utilizing brief information sheet was similar to those that had been used in other large clinical trials such as IST-3 (Third International Stroke Trial).^12^

Most importantly, the use of brief consent appeared acceptable to patients and relatives, as few (2 of 771; 0.3%) withdrew their consent when full consent was requested later. The use of brief consent did not result in higher withdrawal rates from trial or loss to follow-up. The Cord Pilot study, which used a similar 2-stage brief consent, reported positive feedback with participants satisfied with the information received while having sufficient time to make their decision.^13^ Brief consent was also acceptable to clinicians seeking consent.^14^

It is also noteworthy that a larger proportion of patients recruited via 2-stage process were female compared with 1-stage process. While the reasons for this are not known and need to be further explored, the use of 2-stage consent could potentially improve the enrollment of female participants, who are frequently underrepresented in acute stroke trials.^15^

Similar to previous reports,^16,17^ patients recruited via proxy consent were older, had more severe strokes, and would have been excluded from the trial if legal representatives’ consent had not been permitted. Exclusion of incapacitated patients does not only unfairly deprive such patients from receiving potentially beneficial treatment but it also introduces selection bias, as only patients with milder stroke can be recruited. In this respect, the option to include doctors as a professional legal representatives enabled patients who lacked capacity to participate in the trial when no relatives were available. Prior knowledge of the natural history of ICH and the risks and benefits of tranexamic acid combined with the use of an initial brief consent enabled professional legal representative to make enrollment decisions rapidly on behalf of incapacitated patients. However, it needs to be noted that this consent pathway was not permitted in all participating countries.

Proxy consent by a relative was associated with significant delays and markedly reduced odds of randomization within 3 hours of onset compared with self-consent. This supports previous findings that relatives might not always be suitable surrogate decision makers.^18,19^ Furthermore, relatives may not be physically present at the bedside, especially in the COVID-19 era,^20^ and additional time is spent looking for the relatives in a busy emergency department. The use of digital technology via telemedicine, videotelephony, and electronic forms to seek consent from relatives may be an alternative to conventional face-to-face consultations.^21–23^ However, the patient’s relatives may be stressed and distracted and need more time to consider their decisions or may be unable to make decisions.^2,18,24^ Results of a focus group consultation suggest that stroke survivors are worried about the additional stress the consent process imposes on an already distressed relative.^25^

Uncertainties about the patient’s wishes, the complexities of the patient’s condition and intervention, and the use medical terminology may lead to relatives requiring longer time for consent.^26,27^ Although it was reassuring that only 2 patients in this study who regained capacity disagreed with their relatives’ decision and withdrew consent, it remains unclear how much time should be allowed for the consent process in an emergency setting and how much information should be provided.

Current European Union regulations, approved in 2014 after the initiation of the TICH-2 trial, allow recruitment of patients in emergency clinical trials without prior consent, if the patient lacks capacity, and it is not possible to obtain informed consent from a legal representative within the therapeutic window.^28^ This directive defined the concept that expert clinicians and ethics committee, based on rigorous review of study protocol, are in a better position to make decisions on whether the trial was designed to the patient’s best interest.^29^ Furthermore, community consultation during the ethics review process ensures opinions and concerns of the study population are taken into consideration.^22^ While deferral or waiver of consent is permissible in emergency situations and may be a preferable option, many researchers do not utilize this approach. A deferral or waiver of consent was also recommended by the Hemorrhagic Stroke Academia Industry Roundtable and the European Stroke Organisation Trials Network Committee as one approach of reduce time to treatment in stroke trials.^21,30^ Such deferral or waiver of consent can be appropriate when the condition studied is acute, rapidly deteriorating, with poor outcome, and the intervention studied has good safety profile.^22^ Some trialists suggest that seeking consent is unethical if it delays the initiation of trial treatment leading to reduced treatment effects. This is especially so if the trial intervention constitutes the only possibility for an improved outcome.^22,31,32^ Waiver of consent has been successfully applied in several emergency trials, such as FASTEST (FVIIa for Acute Hemorrhagic Stroke Administered at Earliest Time Trial; https://www.clinicaltrials.gov; unique identifier: NCT03496883) and ESETT (Established Status Epilepticus Treatment Trial).^33^

One limitation of this study is that we have not surveyed patients, relatives, or doctors regarding the consent process. Future trials could explore the implications of 2-stage consent on participants’ experience, including if they felt that they were appropriately involved, the quality of interaction with researchers, what they felt was important at the time of decision-making, their perception, understanding of their contribution to research as study participants, recall of the consent process at a later date, and postenrollment discussion. Although withdrawal after initial brief consent was rare, we have not captured data of potentially eligible patients who declined participation when first approached. As TICH-2 had a short enrollment period of only 8 hours, it is uncertain whether a 2-stage consent is appropriate and effective for trials with longer recruitment windows. Doctor consent was only used in a minority (8%) of patients despite a shorter time to enrollment. As it was not a requirement to appoint professional legal representative a priori, it may be difficult to establish in an emergency whether a doctor is truly unconnected to the trial. Brief and doctor consents were not permitted in some countries due to regulatory requirements, limiting the generalizability of their use.

## CONCLUSIONS

In conclusion, offering a 2-stage consent process and engaging doctors as professional legal representatives should be considered in emergency stroke trials with tight recruitment time windows. Both processes appeared acceptable with good follow-up completion and the possibility to recruit more patients with severe ICH.

## Article Information

### Acknowledgments

We thank the patients, investigators, and research staff at the participating sites and members of the Independent Data Monitoring Committee, for their involvement in and support for the study.

### Sources of Funding

TICH-2 (Tranexamic Acid in Intracerebral Haemorrhage-2) was funded by the National Institute for Health Research (NIHR) Health Technology Assessment (NIHR HTA project code 11_129_109).

### Disclosures

P.M. Bath is Stroke Association Professor of Stroke Medicine and a National Institute for Health Research (NIHR) Senior Investigator. He has received consulting fees from DiaMedica, Moleac, Nestle, Phagenesis, and Sanofi; he is an unpaid advisor to Platelet Solutions. Dr Robinson is an NIHR Senior Investigator. R. Collins has accepted speaker’s honoraria from Bayer, Daichii Sankyo, and Pfizer. Dr Werring has received honoraria from Bayer, Alnylam, Portola, and NovNordisk. Dr Al-Shahi Salman has received grants from NIHR during the conduct of the study and grants from the British Heart Foundation outside the submitted work. Dr Appleton, Dr Dineen, N. Sprigg, Dr Roffe, and Dr England report grants from NIHR Health Technology Assessment during the conduct of the study. Dr Lyrer has received advisory board compensation from Bayer, Switzerland; nonfinancial support, advisory board compensation, and travel grant from Boehringer Ingelheim; advisory board compensation and travel grant from Pfizer; research grants from University Hospital Basel, Neurology Clinic; research grants from the Swiss National Foundation SNF; research grants from ACTICOR France; advisory board compensation from Biogen, Switzerland; investigator fees from Alexion; and grants from Bayer, Germany, outside the submitted work. Dr Karlinski has received personal fees from Boehringer Ingelheim, Pfizer, Bayer, and Medtronic outside the submitted work. H. Christensen has received national lead fees to institutional research account from Bayer and Portola and personal fees from Bristol Myers Squibb and Bayer outside the submitted work. L. Duley was the chief investigator of a perinatal trial, which developed a similar brief consent process. This was adapted for use in acute stroke, as reported in the current study.

### Supplemental Material

Supplemental Methods S1–S6

Tables S1–S4

## Supplementary Material


