# Supplementary material for: Brief Consent Methods Enable Rapid Enrollment in Acute Stroke Trial: Results From the TICH-2 Randomized Controlled Trial
Source: Stroke. 2021 Dec 1;53(4):1141–8. doi: 10.1161/STROKEAHA.121.035191 (PMC7612544; doi:10.1161/STROKEAHA.121.035191)
Supplement: Supplementary file 1 [file str-53-1141-s001.pdf]

Supplemental Material
Supplemental Methods I

STROBE Statement—Checklist of items that should be included in reports of ***cohort studies***

	Item No	Recommendation	Page No
Title and abstract	1	(*a*) Indicate the study's design with a commonly used term in the title or the abstract	title page
		(*b*) Provide in the abstract an informative and balanced summary of what was done and what was found	1
Introduction			
Background/rationale	2	Explain the scientific background and rationale for the investigation being reported	3
Objectives	3	State specific objectives, including any prespecified hypotheses	3
Methods			
Study design	4	Present key elements of study design early in the paper	3
Setting	5	Describe the setting, locations, and relevant dates, including periods of recruitment, exposure, follow-up, and data collection	Previously published (Reference 3-5), page 8
Participants	6	(*a*) Give the eligibility criteria, and the sources and methods of selection of participants. Describe methods of follow-up	Previously published (Reference 3-5)
		(*b*) For matched studies, give matching criteria and number of exposed and unexposed	
Variables	7	Clearly define all outcomes, exposures, predictors, potential confounders, and effect modifiers. Give diagnostic criteria, if applicable	7; 4-6, Table 1
Data sources/ measurement	8*	For each variable of interest, give sources of data and details of methods of assessment (measurement). Describe comparability of assessment methods if there is more than one group	7
Bias	9	Describe any efforts to address potential sources of bias	4-5
Study size	10	Explain how the study size was arrived at	Previously published (Reference 3-5)
Quantitative variables	11	Explain how quantitative variables were handled in the analyses. If applicable, describe which groupings were chosen and why	7
Statistical methods	12	(*a*) Describe all statistical methods, including those used to control for confounding	7
		(*b*) Describe any methods used to examine subgroups and interactions	
		(*c*) Explain how missing data were addressed	
		(*d*) If applicable, explain how loss to follow-up was addressed	
		(*e*) Describe any sensitivity analyses	
Results			
Participants	13*	(a) Report numbers of individuals at each stage of study—eg numbers potentially eligible, examined for eligibility, confirmed eligible, included in the study, completing follow-up, and analysed	8, 15, Supplemental Method II, Supplemental Table II
		(b) Give reasons for non-participation at each stage	
		(c) Consider use of a flow diagram	
Descriptive data	14*	(a) Give characteristics of study participants (eg demographic, clinical, social) and information on exposures and potential confounders	Table 2, Table 3 footnote, pg 18-20,

		(b) Indicate number of participants with missing data for each variable of interest<br>(c) Summarise follow-up time (eg, average and total amount)	supplemental Table II
Outcome data	15*	Report numbers of outcome events or summary measures over time	Table 3, pg 21-22, supplemental Table I

Main results	16	(*a*) Give unadjusted estimates and, if applicable, confounder-adjusted estimates and their precision (eg, 95% confidence interval). Make clear which confounders were adjusted for and why they were included<br>(*b*) Report category boundaries when continuous variables were categorized<br>(*c*) If relevant, consider translating estimates of relative risk into absolute risk for a meaningful time period	Table 4, pg 24-25
Other analyses	17	Report other analyses done—eg analyses of subgroups and interactions, and sensitivity analyses	Supplement Table IV
Discussion			
Key results	18	Summarise key results with reference to study objectives	10
Limitations	19	Discuss limitations of the study, taking into account sources of potential bias or imprecision. Discuss both direction and magnitude of any potential bias	13-14
Interpretation	20	Give a cautious overall interpretation of results considering objectives, limitations, multiplicity of analyses, results from similar studies, and other relevant evidence	12
Generalisability	21	Discuss the generalisability (external validity) of the study results	11, 14
Other information			
Funding	22	Give the source of funding and the role of the funders for the present study and, if applicable, for the original study on which the present article is based	14

*Give information separately for exposed and unexposed groups.

Note: An Explanation and Elaboration article discusses each checklist item and gives methodological background and published examples of transparent reporting. The STROBE checklist is best used in conjunction with this article (freely available on the Web sites of PLoS Medicine at http://www.plosmedicine.org/, Annals of Internal Medicine at http://www.annals.org/, and Epidemiology at http://www.epidem.com/). Information on the STROBE Initiative is available at http://www.strobe-statement.org.

Supplemental Method II- Flow diagram

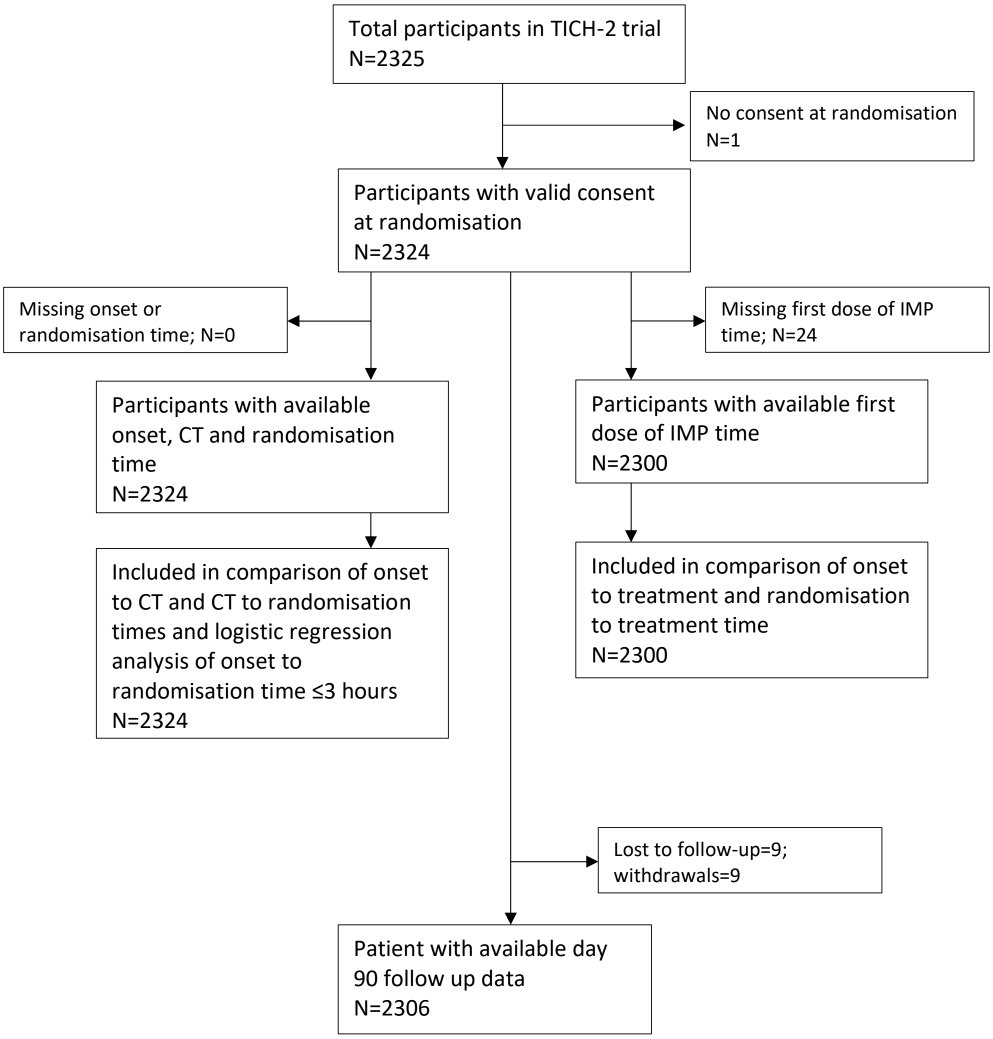

Supplemental Methods III

(Form to be printed on local headed paper)

Patient Information Sheet

(Final version 1.1: 28 February 2014)

Title of Study: Tranexamic acid for Haemorrhage Stroke (TICH2)

REC ref: 12/EM/0369 CTA ref : 2012-004108-37

Name of Researcher:

You have been invited to take part in a research study and it is important for you to understand why the research is being done and what it will involve. We would like you to read the following information carefully. You may talk to others about the study if you wish. We would like you to take as long you need to make your decision however the potential benefits and harms of this treatment may be related to how soon after stroke the treatment is given so we do need to decide about giving the treatment as quickly as possible. Ask us if there is anything that is not clear to you, or if you would like more information. Thank you for reading this.

<u>What is the purpose of the study?</u>

When someone has a stroke caused by bleeding into the brain (haemorrhagic stroke) permanent brain damage can occur and result in long term disability. There is also a chance that the bleeding can increase, which may cause worse disability or be life threatening. This happens in approximately 20-30% of haemorrhagic stroke patients.

At present there is no available treatment that is effective at reducing the bleeding in the brain and improving the recovery. New treatments are being developed to treat stroke, but it can be very hard to test whether they work in the first few hours because often patients take longer than this to get to hospital and have investigations such as brain scanning. Also some treatments are not suitable for all patients. This can make testing new treatments difficult.

In this trial, we want to test whether it is possible to give a drug (tranexamic acid) to patients in the first few hours after a haemorrhagic stroke. We hope that we will be able to show that giving the drug may reduce the chances of dying and being left with disability after a haemorrhagic stroke.

In this trial, the treatment we are testing is a drug that encourages blood to clot to stop bleeding. Continued or increased bleeding into the brain (so called haematoma expansion) is not uncommon in the first hours and days following a haemorrhagic stroke and increases the risk of the patient not recovering fully and being left with some disability. Stopping the bleeding in the first hours and days after stroke with medications might help patients to recover better. This drug is not given routinely after stroke.

We aim to assess in this trial what effect tranexamic acid has on blood after a haemorrhagic stroke. Tranexamic acid is a tried and tested drug in other medical conditions that acts quickly to help the blood to clot and stop bleeding.

In order to do a proper comparison we need to give some people the active drug and some people a dummy (placebo) treatment. In this trial the dummy treatment is salt water and half of the patients in the trial will receive an injection with the drug tranexamic acid and half will have an injection of salt water as a dummy (placebo) treatment.

The data will help doctors decide whether blood thickening treatments like tranexamic acid can be used in patients with acute haemorrhagic strokes to try to prevent death and improve recovery.

<u>Why have I been chosen?</u>

You have been chosen because unfortunately you have had a stroke caused by bleeding into the brain – this is called a haemorrhagic stroke.

<u>Do I have to take part in the study?</u>

It is up to you to decide whether or not you take part in the study. If you do decide to take part you will be given this information sheet to keep and be asked to sign a consent form. You are still free to withdraw at any time without giving a reason.

Deciding you do not want to take part in the study will not affect the standard of care you receive.

<u>What will happen to me if I take part?</u>

Your involvement in the study will last for 12 months. In this study the treatment (either tranexamic acid or dummy) is given as an injection via a drip which will be given over approximately 8 hours.

We select which treatment you receive randomly (like tossing a coin) because this is how most clinical trials are carried out. When we don't know whether a treatment is effective or not we need to test it against not getting that treatment (called a control). Using randomisation to put patients into treatment groups is the best way to get a true answer as to whether a treatment works or not. In this trial it is important to test whether the drug tranexamic acid can be given at random in a trial. Half the participants will receive the drug tranexamic acid injection and half will have an injection of salt water as a dummy (placebo) treatment.

The treatment (either tranexamic acid or dummy) will be given as an injection as soon as possible once you have decided you wish to take part in the study. The treatment will be given via a drip over approximately 8 hours. You will not know if you received the drug or the dummy. The treatment will be given once, and then the treatment will stop.

During the next 7 days a nurse will check your condition looking in particular for signs of side effects of the treatment. We will also repeat a brain scan the day after the treatment to assess effects of the treatment. The brain scan will last less than 5 minutes but you will need to go to the x-ray department for the brain scan which may take approximately an hour.

We ask you permission to contact your GP or check with the NHS Information Centre to check on your condition three and twelve months after your stroke and to confirm your contact details. You will then be contacted for a telephone consultation with a member of the research team. This is to check your condition at that time. It will involve asking how

you are able to move around, about how you feel your life has been affected by the stroke and some brief memory tests. In order to make the final evaluation of the study as objective as possible, the person who telephones you will not know if you received the active treatment or not.

Other than described here, your treatment will be exactly the same as for all stroke patients.

<u>Expenses and payments</u>

You will not receive payment for participating in this study. There will be no charge for the trial medication.

<u>What is the drug, device or procedure that is being tested?</u>

Tranexamic acid is a medicine which has been used for many years to treat bleeding problems. It is currently used in patients with nose bleeds, bleeding from the bladder or womb and heavy bleeding after surgical operations or after trauma. It increases blood clotting quickly and effectively for short periods in patients with a bleeding condition and that is why we are using it in this study.

<u>What are the alternatives for diagnosis or treatment?</u>

There are currently no licensed medications used to reverse or stop bleeding after a haemorrhagic stroke.

We do know that all patients with stroke benefit from care on an acute stroke unit. You do not need to take part in this study to receive standard acute stroke care.

If you decide to take part in the study, this will not affect your right to receive appropriate medical care from your doctor.

<u>What are the side effects of any treatment received when taking part?</u>

Treatment with any drugs can result in possible side effects and the side effects from tranexamic acid are generally mild. They can include diarrhoea, low blood pressure and dizziness. The drug can also sometimes affect colour vision but this is rare.

However, because the treatment works by stopping bleeding there is a chance it can cause an increase in blood clot formation. This can occur in the legs (deep vein thrombosis, DVT) or the lungs (Pulmonary embolism, PE) and is potentially very serious and maybe even life threatening. If you have previously suffered from blood clots in the legs or lungs you may not be able to participate in this study.

In a very large study in 20,000 people with serious bleeding, tranexamic acid was safe and reduced the number of people dying from bleeding. There was no increase in serious side effects, such as blood clots, in the patients who were treated with tranexamic acid.

Because tranexamic acid is already routinely used in a number of bleeding conditions, we expect the potential benefit of the drug (stopping bleeding in to the brain) to outweigh the low risk of serious side effects (such as blood clots). However we do not know this for certain and will monitor all participants closely for side effects.

You must inform your doctor or member of the research team if you feel you have had a reaction to the medication.

What are the other possible disadvantages and risks of taking part?

Your will have an extra CT brain scan performed as part of this trial. This is exactly the same as the CT scan that you had when you first came to hospital. The scan itself takes less than 5 minutes and does not involve any injections. The scan uses x-rays, which in large amounts can be harmful, but for this extra CT head scan the additional risk to you from the scan has been judged to be extremely small and is comparable with the annual risk of dying from an accident in the home.

You will need to be followed up by the research team for 12 months after starting the study.

What are the possible benefits of taking part?

Your participation in this study may reduce the symptoms of your haemorrhagic stroke or improve long-term recovery. However, we cannot promise the study will help you, and participation is voluntary. The information we get from your involvement may benefit other people who may have a stroke in the future.

What happens when the research study stops?

We aim to treat 2000 patients in this study from the UK and other countries. When it has finished we will look at the data and decide whether the treatment has been possible in patients with haemorrhagic stroke. We may not directly tell you or your relative the results of the study, but they will be published in a journal where they can be read.

Will my taking part in the study be kept confidential?

If you join the study, relevant sections of your medical notes and data collected in the study may be looked at by authorised individuals from the University of Nottingham, the research group and regulatory authorities where it is relevant to this study.

They may also be looked at by representatives of regulatory authorities and by authorised people from the Trust, or other NHS bodies to check that the study is being carried out correctly. All will have a duty of confidentiality to you as a research participant and nothing that could reveal your identity will be disclosed outside the research site.

Our procedures for handling, processing, storage and destruction of patient's data are compliant with the Data Protection Act 1998.

In order to be able to contact you about your health, your name and contact details will be made available to the researchers running this study and held at the co-ordinating centre in Nottingham, and not just held by your local study doctor. These details will be kept securely, with access restricted.

Contact Details:

If you have any questions or concerns do not hesitate to contact your local research team on: XXXXXX.

What if relevant new information becomes available?

Sometimes during the course of a research project new information becomes available about the treatment/drug that is being studied. If this happens, your research doctor will tell you about it and discuss whether you want to continue in the study, although after the first 3 days you will no longer be receiving tranexamic acid. If you decide that you do not wish to carry on, the research doctor will make arrangements for your care to continue. If you decide that you wish to continue in the study you will be asked to sign an updated consent form.

Also, on receiving new information the research doctor might consider it to be in your best interests to withdraw them from the study. They will explain the reasons and arrange for your care to continue.

If the study is stopped for any other reason, you will be told why and your continuing care will be arranged.

What will happen if I don't want to carry on with the study?

If you withdraw from the study, we would like to use the data collected up to your withdrawal and will not be able to delete any of the confidential research information we have collected prior to your withdrawal. Your name and contact details will be deleted.

What if there is a problem?

If you had a reaction to the medication we would stop the medication and appropriate medical care would be given. Any adverse events are monitored.

Complaints

If you have a concern about any aspect of this study, you should ask to speak with the local researcher team who will do their best to answer your questions on XXXXXX. If you remain unhappy and wish to complain formally, you can do this through your hospital's NHS Complaints Procedure.

Harm

If you are harmed during the research and this is due to someone's negligence then you may have grounds for legal action and compensation, but you may have to pay your legal costs. The normal hospital complaints mechanisms will be available to you. If you are harmed during research and this is not due to someone's negligence then there are no special compensation arrangements, but you may still follow the normal hospital complaints mechanisms. Again you may have to pay your legal costs. The University of Nottingham maintains Clinical Trials Insurance to cover the University's legal liability.

Involvement of the General Practitioner/Family doctor (GP)

If you are enrolled in the study we will inform your General Practitioner as a matter of courtesy.

What will happen to the results of the research study?

The results of the research may be published. If so, this will be in a medical journal. You will not be identified in any report.

Who is organising and funding the research?

The University of Nottingham is sponsor for the study.

Who has reviewed the study?

The [NAME] research ethics committee has reviewed the research.

Supplemental Methods IV

(Form to be printed on local headed paper)

Relative Information Sheet

(Final version 1.1: 28 February 2014)

Title of Study: Tranexamic acid for Haemorrhage Stroke (TICH2)

REC ref: 12/EM/0369 CTA ref : 2012-004108-37
Name of Researcher:

Your relative or close friend has been invited to take part in a research study. The study is testing whether the drug treatment tranexamic acid can improve outcome after stroke caused by bleeding into the brain. We would normally ask them if they wish to take part, however as they are currently unable to make a decision on their own about whether or not to enter the trial themselves, we need to gather more information about their wishes and feelings.

We would like you to decide whether you feel your relative would wish to participate in the trial were they able to decide for themselves. It is important for you to understand why the research is being done and what it will involve. We would like you to read the following information carefully. You may talk to others about the study if you wish. We would like you to take as long you need to make your decision however the potential benefits and harms of this treatment may be related to how soon after stroke the treatment is given so we do need to decide about giving the treatment as quickly as possible. Ask us if there is anything that is not clear to you, or if you would like more information. Thank you for reading this.

What is the purpose of the study?

When someone has a stroke caused by bleeding into the brain (haemorrhagic stroke) permanent brain damage can occur and result in long term disability. There is also a chance that the bleeding can increase, which may cause worse disability or be life threatening. This happens in approximately 20-30% of haemorrhagic stroke patients.

At present there is no available treatment that is effective at reducing the bleeding in the brain and improving the recovery. New treatments are being developed to treat stroke, but it can be very hard to test whether they work in the first few hours because often patients take longer than this to get to hospital and have investigations such as brain scanning. Also some treatments are not suitable for all patients. This can make testing new treatments difficult.

In this trial, we want to test whether it is possible to give a drug (tranexamic acid) to patients in the first few hours after a haemorrhagic stroke. We hope that we will be able to show that giving the drug may reduce the chances of dying and being left with disability after a haemorrhagic stroke.
In this trial, the treatment we are testing is a drug that encourages blood to clot to stop bleeding. Continued or increased bleeding into the brain (so called haematoma expansion) is not uncommon in the first hours and days following a haemorrhagic stroke and increases the risk of the patient not recovering fully and being left with some

disability. Stopping the bleeding in the first hours and days after stroke with medications might help patients to recover better. This drug is not given routinely after stroke.

We aim to assess in this trial what effect tranexamic acid has on blood after a haemorrhagic stroke. Tranexamic acid is a tried and tested drug in other medical conditions that acts quickly to help the blood to clot and stop bleeding.

In order to do a proper comparison we need to give some people the active drug and some people a dummy (placebo) treatment. In this trial the dummy treatment is salt water and half of the patients in the trial will receive the drug tranexamic acid injection and half will have an injection of salt water as a dummy (placebo) treatment.

The data will help doctors decide whether blood thickening treatments like tranexamic acid can be used in patients with acute haemorrhagic strokes to try to prevent death and improve recovery.

<u>Why has my relative been chosen?</u>

Your relative has been chosen because unfortunately they have had a stroke caused by bleeding into their brain – this is called a haemorrhagic stroke.

We are asking you to make the decision as to whether your relative should take part in the trial as their illness prevents them from deciding for themselves.

<u>Do they have to take part in the study?</u>

It is up to you to decide whether or not your relative should take part in the study. If you do decide they will take part, you will be given this information sheet to keep and be asked to sign a proxy consent form. You are still free to withdraw your relative at any time without giving a reason. If your relative's medical condition improves so that they are able to decide for themselves, they will be given information on the study and asked if they wish to continue. They may decide to withdraw from the study at any time.

Deciding you do not want your relative to take part in the study will not affect the standard of care your relative receives.

<u>What will happen to my relative if they take part?</u>

Their involvement in the study will last for 12 months. In this study tranexamic acid is given as an injection into a small plastic tube (cannula) inserted into a vein, in the same way that drugs are given via a drip.

We select which treatment they receive randomly (like tossing a coin) because this is how most clinical trials are carried out. When we don't know whether a treatment is effective or not we need to test it against not getting that treatment (called a control). Using randomisation to put patients into treatment groups is the best way to get a true answer as to whether a treatment works or not. In this trial it is important to test whether the drug tranexamic acid can be given at random in a trial. . Half the participants will receive the drug tranexamic acid injection and half will have an injection of salt water as a dummy (placebo) treatment.
The treatment (either tranexamic acid or dummy) will be given as an injection as soon as possible once you have decided you wish your relative to take part in the study. The treatment will be given via a drip over approximately 8 hours. They will not know if they

received the drug or the dummy. The treatment will be given once and then the treatment will stop.

During the next 7 days a nurse will check your relative's condition looking in particular for signs of side effects of the treatment. We will also repeat a brain scan the day after the treatment to assess effects of the treatment. The brain scan will last less than 5 minutes but they will need to go to the x-ray department for the brain scan which may take approximately an hour.

We ask your permission to contact your relative's GP or check with the NHS Information Centre to check on your relative's condition three and twelve months after their stroke and to confirm their contact details. Your relative will then be contacted for a telephone consultation with a member of the research team. This is to check their condition at that time. It will involve asking how well they are able to move around, about how they feel their life has been affected by the stroke and some brief memory tests. In order to make the final evaluation of the study as objective as possible, the person who telephones your relative will not know if they received the active treatment or not.

Other than described here, your relative's treatment will be exactly the same as for all stroke patients.

Expenses and payments

Your relative will not receive payment for participating in this study. There will be no charge for the trial medication.

What is the drug, device or procedure that is being tested?

Tranexamic acid is a medicine which has been used for many years to treat bleeding problems. It is currently used in patients with nose bleeds, bleeding from the bladder or womb and heavy bleeding after surgical operations or trauma. It increases blood clotting quickly and effectively for short periods in patients with a bleeding condition and that is why we are using it in this study.

What are the alternatives for diagnosis or treatment?

There are currently no licensed medications used to reverse or stop bleeding after a haemorrhagic stroke.

We do know that all patients with stroke benefit from care on an acute stroke unit. Your relative does not need to take part in this study to receive standard acute stroke care.

If you decide that your relative will take part in the study, this will not affect their right to receive appropriate medical care from their doctor.

What are the side effects of any treatment received when taking part?

Treatment with any drugs can result in possible side effects and the side effects from tranexamic acid are generally mild. They can include diarrhoea, low blood pressure and dizziness. The drug can also sometimes affect colour vision but this is rare.
However, because the treatment works by stopping bleeding there is a chance it can cause an increase in blood clot formation. This can occur in the legs (deep vein thrombosis, DVT) or the lungs (Pulmonary embolism, PE) and is potentially very serious and maybe

even life threatening. If your relative has previously suffered from blood clots in the legs or lungs they may will not be able to participate in this study.

In a very large study in 20,000 people with serious bleeding, tranexamic acid was safe and reduced the number of people dying from bleeding. There was no increase in serious side effects, such as blood clots, in the patients who were treated with tranexamic acid.

Because tranexamic acid is already routinely used in a number of bleeding conditions, we expect the potential benefit of the drug (stopping bleeding in to the brain) to outweigh the low risk of serious side effects (such as blood clots). However we do not know this for certain and will monitor all participants closely for side effects.

If you feel your relative has had a reaction to the medication please inform a member of the research team or your relative's clinical team (doctor or nurse) who will inform the research team.

What are the other possible disadvantages and risks of taking part?

Your relative will have an extra CT brain scan performed as part of this study. This is exactly the same as the CT scan that they had when they first came to hospital. The scan itself takes less than 5 minutes and does not involve any injections. The scan uses x-rays, which in large amounts can be harmful, but for this extra CT head scan the additional risk to your relative from the scan has been judged to be extremely small, comparable with the annual risk of dying from an accident in the home.

Your relative will need to be followed up by the research team for 12 months after starting the study.

What are the possible benefits of taking part?

Your relative's participation in this study may reduce the symptoms of their haemorrhagic stroke or improve long-term recovery. However, we cannot promise the study will help your relative, and participation is voluntary. The information we get from your relative's involvement may benefit other people who may have a stroke in the future.

What happens when the research study stops?

We aim to treat 2000 patients in this study from the UK and other countries. When it has finished we will look at the data and decide whether the treatment has been possible in patients with haemorrhagic stroke. We may not directly tell you or your relative the results of the study, but they will be published in a journal where they can be read.

Will my relatives taking part in the study be kept confidential?

If your relative joins the study, relevant sections of their medical notes and data collected in the study may be looked at by authorised individuals from the University of Nottingham, the research group and regulatory authorities where it is relevant to this study.

They may also be looked at by representatives of regulatory authorities and by authorised people from the Trust, or other NHS bodies to check that the study is being carried out correctly. All will have a duty of confidentiality to the research participant and nothing that could reveal their identity will be disclosed outside the research site.

Our procedures for handling, processing, storage and destruction of patient's data are compliant with the Data Protection Act 1998.

In order to be able to contact your relative's general practitioner their name and contact details will be made available to the researchers running this study and held at the co-ordinating centre in Nottingham, and not just held by your relative's local study doctor. These details will be kept securely, with access restricted.

<u>Contact Details:</u>

If you have any questions or concerns do not hesitate to contact your local research team on: xxxxxxx.

<u>What if relevant new information becomes available?</u>

Sometimes during the course of a research project new information becomes available about the treatment/drug that is being studied. If this happens, your research doctor will tell you about it and discuss whether you want your relative to continue in the study, although after the first 3 days they will no longer be receiving tranexamic acid. If you decide that your relative should not wish to carry on, the research doctor will make arrangements for their care to continue. If you decide that your relative can continue in the study you will be asked to sign an updated assent form if they are still unable to do so themselves.

Also, on receiving new information the research doctor might consider it to be in your relative's best interests to withdraw them from the study. They will explain the reasons and arrange for their care to continue.

If the study is stopped for any other reason, you will be told why and your relative's continuing care will be arranged.

<u>What will happen if I don't want my relative to carry on with the study?</u>

You can withdraw your relative anytime from the study without having to give any reason. We would like to use the data collected up to their withdrawal and will not be able to delete any of the confidential research information we have collected prior to their withdrawal. Their name and contact details will be deleted.

<u>What if there is a problem?</u>

If your relative has a reaction to the medication we would stop the medication and appropriate medical care would be given. Any adverse events are monitored.

<u>Complaints</u>

If you have a concern about any aspect of this study, you should ask to speak with the local researchers who will do their best to answer your questions on XXXXX. If you remain unhappy and wish to complain formally, you can do this through the hospital's NHS Complaints Procedure.

<u>Harm</u>

In the event that something does go wrong and your relative is harmed during the research study there are no special compensation arrangements. If they are harmed and this is due to someone's negligence then you may have grounds for a legal action for compensation but you/your relative may have to pay the legal costs.

Involvement of the General Practitioner/Family doctor (GP)

If your relative is enrolled in the study we will inform their General Practitioner as a matter of courtesy.

What will happen to the results of the research study?

The results of the research may be published. If so, this will be in a medical journal. Your relative will not be identified in any report.

Who is organising and funding the research?

The University of Nottingham is sponsor for the study.

Who has reviewed the study?

The [NAME] research ethics committee has reviewed the research.

Supplemental Methods V

(Form to be printed on local headed paper)

Brief Patient Information Sheet

(Final version 1.0: 19 November 2012)

Title of Study: Tranexamic acid for Haemorrhage Stroke (TICH2)

REC ref: 12/EM/0369 CTA ref : 2012-004108-37

Name of Researcher:

"You have had a haemorrhagic stroke (a stroke caused by bleeding in the brain) that needs urgent care. You will get all the usual emergency care for stroke that we provide at this hospital. As well as this, we would like to include you in an international study. This study will see whether a drug called tranexamic acid reduces bleeding inside the head after haemorrhagic stroke. We hope that the drug will lead to a better recovery. We know that the drug reduces bleeding in other types of haemorrhage conditions, without side effects, and the sooner the drug is given the more effective it is. As yet we don't know if it works in haemorrhagic stroke.

As part of the study, you will receive an injection into a vein followed by a drip over eight hours. Half the patients in the study will get the tranexamic acid and half a dummy drug (a liquid which does not contain tranexamic acid). We won't know until the end of the study who received which treatment.

We will need to collect some information about your medical condition and send it to a central office in Nottingham. We would also like to perform one additional CT brain scan (similar to the one you had to diagnose your stroke) to monitor the effect of the treatment.

If you would like to know more about our study now, then we will tell you. But otherwise we will tell you more about it later.

Are you happy for us to go ahead with the study treatment?"

Yes, I am happy for you to go ahead.

Name: ______________________________________

Signature: __________________________________

Date: ____________________

Witness name and signature:

Witness Name: ______________________________________

Witness Signature: __________________________________

Date: ____________________

(Complete only where required)

Supplemental Methods VI

(Form to be printed on local headed paper)

Brief Relative Information Sheet

(Final version 1.0: 19 November 2012)

Title of Study: Tranexamic acid for Haemorrhage Stroke (TICH2)

REC ref: 12/EM/0369 CTA ref : 2012-004108-37

Name of Researcher:

"Your relative has a haemorrhagic stroke (a stroke caused by bleeding in the brain) that needs urgent care. He/she will get all the usual emergency care for stroke that we provide at this hospital. As well as this, we would like to include him/her in an international study. This study will see whether a drug called tranexamic acid reduces bleeding inside the head after haemorrhagic stroke. We hope that the drug will lead to a better recovery. We know that the drug reduces bleeding in other types of severe injury and without side effects, but as yet we don't know if it works in haemorrhagic stroke.

As part of the study, your relative will receive an injection into a vein followed by a drip over eight hours. Half the patients in the study will get the tranexamic acid and half a dummy drug (a liquid which does not contain tranexamic acid). We won't know until the end of the study who received which treatment.
We will need to collect some information about your relative's medical condition and send it to a central office in Nottingham. We would also like to perform one additional CT brain scan (similar to the one your relative had to diagnose your stroke) to monitor the effect of the treatment.

If you would like to know more about our study now, then we will tell you. But otherwise we will tell you more about it later.

Are you happy for us to go ahead with the study treatment?"

Yes, I am happy for you to go ahead.

Name: ____

Signature: _____________________________________

Relationship to Patient: ___________________________

Date: ______________________

Witness name and signature:

Witness Name: ____

Witness Signature: ______________________________________

Date: ______________________
(Complete only where required)

Supplemental Tables

Table I Brief consent form use and subsequent full written consent status

TICH-2 Brief consent	All	Patient consent	Relative consent	Doctor consent
Brief consent given at randomisation	771	260	323	188
Follow-on consent by discharge	666 (86.4%)	241 (92.7%)	292 (90.4%)	133 (70.7%)
No follow-on consent by discharge	105 (13.6%)	19 (7.3%)	31 (9.6%)	55 (29.3%)
Reasons no written consent obtained by the time of discharge				
Died	38 (36.2%)	5 (26.3%)	17 (54.8%)	16 (29.1%)
Patient lacked capacity / no relatives available	38 (36.2%)	6 (31.6%)	6 (19.4%)	26 (47.3%)
Patient discharged before written consent could be obtained	8 (7.6%)	3 (15.8%)	2 (6.5%)	3 (5.5%)
Repatriated	13 (12.4%)	3 (15.8%)	3 (9.7%)	7 (12.7%)
Withdrawn / refused further consent	2 (1.9%)	0 (0)	2 (6.5%)	0 (0)
No reason given	6 (5.7%)	2 (10.5%)	1 (3.2%)	3 (5.5%)

Table II Withdrawn, lost to follow-up, patient completing Day 90 follow-up, by consent type

		Consent pathways					
Patient's status at day 90	2324	One-stage patient	Two-stage patient	One-stage relative	Two-stage relative	Two-stage doctor	P-value
Follow-up completed by patients	948	372 (66.8%)	144 (55.4%)	319 (32.0%)	71 (22.0%)	42 (22.3%)	<0.001
Follow-up completed by carer	839	140 (25.1%)	78 (30.0%)	407 (40.9%)	143 (44.3%)	71 (37.8%)	<0.001
Status obtained from GP	20	5 (0.9%)	2 (0.8%)	8 (0.8%)	3 (0.9%)	2 (1.1%)	0.996
Died	499	36 (6.5%)	33 (12.7%)	257 (25.8%)	103 (31.9%)	70 (37.2%)	<0.001
Withdrawn	9*	3 (0.5%)	2 (0.8%)	0 (0.0%)	2 (0.6%)	2 (1.1%)	0.097
Lost to follow up	9*	1 (0.2%)	1 (0.4%)	5 (0.5%)	1 (0.3%)	1 (0.5%)	0.892

* Day 90 modified Rankin scale not available in 18 patients who withdrawn and lost to follow-up.

Table III Comparison of participants randomised within and after 3 hours

Variables	Onset-randomisation ≤3 hours (833, 35.8%)	Onset-randomisation >3 hours (1492, 64.2%)	p
Age (years)	68.3 (13.6)	69.2 (13.9)	0.15
Sex (male)	477 (57.3%)	824 (55.2%)	0.34
Pre-stroke modified Rankin Scale (/5)	0.0 [0.0, 1.0]	0.0 [0.0, 1.0]	0.084
Glasgow coma scale (/15)	15.0 [12.0, 15.0]	14.0 [12.0, 15.0]	0.69
NIHSS score [/42]	14.0 [8.0, 19.5]	11.0 [6.0, 18.0]	<0.001
Aphasia-severe or mute	247 (29.7%)	411 (27.5%)	0.28
Right-sided limb weakness	421 (50.2%)	744 (49.9%)	0.76
Systolic blood pressure (mmHg)	178.9 (29.9)	172.5 (29.5)	<0.001
Haematoma volume (mL)	24.6 (27.3)	23.7 (27.1)	0.44
Intraventricular haemorrhage	264 (31.9%)	443 (30.2%)	0.40
Onset-to-CT time (minutes)	84.8 (25.7)	165.8 (83.1)	<0.001
UK sites	722 (86.7%)	1188 (79.6%)	<0.001
Doctor consent	532 (63.9%)	975 (65.3%)	0.50
Relative consent	432 (59.0%)	887 (63.2%)	0.060
Two-stage consent	343 (41.2%)	428 (28.7%)	<0.001

NIHSS- National Institute of Health Stroke Scale

Table IV Multivariable logistic regression of factors associated with time to randomisation of ≤3 hours, excluding countries which did not recruit participants using two-stage consent (Spain and Hungary)

Variables	Model 1, aOR (95%CI)	P	Model 2, aOR (95%CI)	P	Model 3, aOR (95%CI)	P
Age (years)	1.01 (0.99-1.01)	0.28	1.01 (1.00-1.02)	0.27	1.00 (0.98-1.01)	0.55
Sex (male)	1.23 (0.98-1.54)	0.077	1.22 (0.94-1.58)	0.14	1.11 (0.87-1.40)	0.40
Systolic BP (mmHg)	1.01 (1.00-1.01)	0.001	1.01 (1.00-1.01)	<0.001	1.01 (1.00-1.01)	0.001
NIHSS (/42)	1.02 (1.00-1.03)	0.033	1.02 (1.00-1.04)	0.034	1.04 (1.02-1.06)	<0.001
Onset-to-CT time (minutes)	0.97 (0.96-0.97)	<0.001	0.97 (0.96-0.97)	<0.001	0.97 (0.96-0.97)	<0.001
Recruitment from UK	2.65 (1.94-3.62)	<0.001	3.06 (2.20-4.26)	<0.001	3.27 (2.34-4.56)	<0.001
Consent type*						
2-stage vs 1-stage (reference)	1.88 (1.49-2.38)	<0.001	-		-	-
Doctor vs Self (reference)	-	-	2.26 (1.50-3.43)	<0.001	-	-
Relative vs Self (reference)	-	-	-	-	0.10 (0.03-0.35)	<0.001
